# Self-reported sleepiness in the context of fitness-to-drive

**DOI:** 10.1007/s11325-019-01810-w

**Published:** 2019-03-19

**Authors:** Aanuolupo Ayeni, Gurpreet Singh Beghal, Martino F Pengo, Nimish Shah, Joerg Steier

**Affiliations:** 1 Lane Fox Respiratory Unit / Sleep Disorders Centre, Guy’s and St Thomas’ NHS Foundation Trust, Westminster Bridge Road, London, SE1 7EH UK; 2 Faculty of Life Sciences and Medicine, King’s College London, London, UK; 3 Sleep Disorder Centre, Department of Cardiovascular, Neural and Metabolic Sciences, IRCCS Istituto Auxologico Italiano, Milan, Italy; 4 Jaslok Hospital and Research Centre, Mumbai, India

**Keywords:** Excessive daytime sleepiness, Epworth scale, Driving, Sleep Apnoea

## Abstract

**Background:**

Excessive daytime sleepiness (EDS) is a contributing factor to road traffic accidents. It is commonly assessed using self-administered questionnaires. These assessments are important information when discussing with the Driver and Vehicle Licensing Agency (DVLA) about fitness-to-drive. We hypothesised that patients may be confounded in their assessments after being informed about these potential implications.

**Patients and methods:**

This was a prospective single-centre study. Patients attending clinics for sleep-disordered breathing were asked to fill in the Epworth Sleepiness Scale (ESS) and the Stanford Sleepiness Scale (SSS). Following their consultation, patients were informed about EDS in the context of driving and that the DVLA might request information based on their self-assessed sleepiness. They were then asked to complete the same questionnaires again. Parameters recorded included age, gender, body mass index (BMI), driving licence holder, and collar size. An ESS score above 10 points was defined as EDS.

**Results:**

One hundred twenty-two subjects were studied (age 59.4 years (15.2); 72 males; BMI 32.1 kg/m^2^ (8.3), driving licence held for 25.2 years (20.6) (*n* = 94); collar size 42.7 cm (5.0)). There was no difference in the ESS [8 (8) vs 8 (8) points; *p* = 0.289] or the SSS [2 (2) vs 2 (2) points; *p* = 0.320] between the two occasions, although seven patients (5.7%) changed their scores from “sleepy” to “non-sleepy” and four patients (3.3%) from “non-sleepy” to “sleepy”.

**Conclusion:**

Providing patients with information about the risk of driving in the context of sleepiness does not significantly change how they score their symptoms using self-administered questionnaires; only about 9.0% of the patients had inconsistent results.

## Introduction

Excessive daytime sleepiness (EDS) is one of the cardinal symptoms of patients presenting to sleep laboratories as many patients with EDS suffer from obstructive sleep apnoea syndrome (OSAS), obesity hypoventilation syndrome (OHS), narcolepsy or idiopathic hypersomnia [1, 2]. Severe EDS impacts on behavioural, physiological and cognitive functioning, and it affects quality of life; it results in reduced reaction time, vigilance, alertness, concentration and, subsequently, results in an impaired ability to successfully carry out attention-based activities [3]. EDS while driving is increasingly being recognised as a cause of road traffic accidents (RTA) [4], and approximately 20% of RTAs in the UK are caused by EDS and driver fatigue [5].

It is difficult to objectively assess EDS, but questionnaires like the Epworth Sleepiness Scale (ESS) or the Stanford Sleepiness Scale (SSS) are widely used in clinical practice to subjectively quantify and determine whether an individual is suffering from EDS [1, 2]. However, it is important to recognise that information contained in these tools might be used by the Driver and Vehicle Licensing Agency (DVLA) when deciding on the fitness to drive; drivers who suffer with conditions causing EDS need to cease driving and inform the DVLA [6].

However, patients might offer a biased view when self-reporting their symptoms by using tools like the ESS, if they know about possible implications regarding their driving licence. We hypothesised that patients might change how they score on the ESS after they are made aware of official guidance regarding fitness-to-drive.

## Patients and methods

### Compliance with ethical standards

This was a prospective study conducted at a clinic for sleep-disordered breathing at a tertiary university hospital between June 2017 and July 2017 (registration number: GSTT/2017/7478). Patients were informed and consented prior to their clinic appointment, and adults aged 18 years and above were included. Patients were addressed following initial identification by the direct clinical care team. Funding was provided via the King’s Undergraduate Research Fellowship 2017, King’s College London, UK (A.A.). The authors have no conflict of interest related to the content of the manuscript.

### Inclusion and exclusion criteria

The following inclusion criteria were assessed prior to inclusion into the analysis:

- Patient referred to the sleep centre
- Aged > 18 years
- Both genders
- Literate and holding capacity
- Fluent English speaker.

In addition, patients were excluded if they met any of the below exclusion criteria:

- Aged <18 years
- Unable to read or write, illiterate
- Non-English speakers
- Not holding capacity
- Acute illness or delirium.

### Short protocol

Parameters recorded included age, gender, ethnicity, body mass index (BMI), past medical history, drug history, allergies, driving licence holder (in years), number of previous road traffic accidents (RTA), smoking (in pack years), alcohol consumption (in units/week), use of illegal highs/illicit substances and collar size.

The first ESS and the SSS were filled in prior to the consultation with the sleep physician. Following the consultation, patients were informed about the risk of EDS and driving, and about official DVLA guidance. It was pointed out that their self-assessed sleepiness might provide information for the DVLA to decide about their fitness-to-drive. Prior to completing a second ESS and the SSS, patients were read the following statement:

If an individual has any condition that affects their ability to drive, which lasts longer than three months, they must inform the DVLA. The DVLA then considers whether sleepiness influences the ability to drive when reviewing these cases. [7]

All appointments were timed to be 15–30 min slots. The 1st questionnaires were filled in prior to and the 2nd questionnaires following these slots. All patients completed the entire assessment within a 60-min time frame.

### Epworth sleepiness scale

The ESS is a questionnaire with eight items to measure severity of daytime sleepiness; respondents report their likelihood of falling asleep in situations using a 4-point Likert scale (‘0’ not at all to ‘3’ highly likely). Responses are summed and higher scores indicate greater sleepiness, the minimum total score is 0 and the highest ‘24’ points; scores of more than 10 points suggest excessive daytime sleepiness [8].

### Stanford Sleepiness Scale

The SSS is a self-rated 7-point scale of equal intervals to quantify the symptom [9]. The scale is a one-item questionnaire measuring levels of sleepiness throughout the day. It is generally used to track alertness at different hours of the day and ranges from ‘feeling active and vital; alert, wide awake’ (1) to ‘Asleep’ (x) and is widely used to assess the effects of sleep deprivation. A score of more than 3 points at any time when the respondent should be feeling alert indicates serious sleep debt [10].

### Outcome parameters

The primary outcome of this study was the change in the ESS (ΔESS, points). The secondary outcome parameters were the change in the SSS (ΔSSS, points), factors associated with a change in the ESS (sleepiness, driving licence holder, RTA) and patients who changed from ‘sleepy’ (ESS > 10) to ‘non-sleepy’ self-assessment (*n*).

### Sample size calculation

**Fig. 1:**
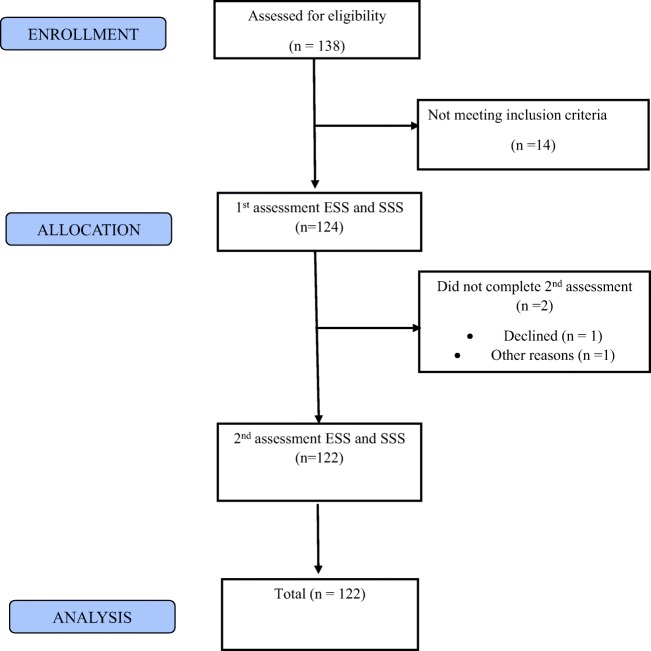
Modified CONSORT diagram. Fourteen patients were excluded, as they were unable to communicate sufficiently in English

The sample size was calculated based on the change in the ESS. Expecting a change in the ESS of two or more points in the total score with an alpha of 5%, a power of 90%, and an approximate standard deviation of the mean difference of the paired observation of three points, a total sample size of 122 patients would need to be included [11]. Following previous experience with the dropout of patients who were assessed in similar scenarios [10], we expected that an additional 5–10% would need to be recruited. Finally, we addressed 138 patients, 14 did not meet inclusion criteria and 2 patients did not complete the assessment; a complete dataset was included for analysis in 122 patients (Fig. 1).

### Statistical analysis

The statistical analysis was conducted using SPSS V23.0 (IBM Corp, Armonk, NY/USA). The Shapiro-Wilk normality test was used to assess data distribution. Results from the ESS, the SSS and subgroups of patients based on EDS (ESS > 10), and whether they held a driving licence were compared using the Wilcoxon signed ranks *t* test. ESS and SSS scores are presented as median (interquartile range) and age, gender, BMI, driving licence duration, smoking history and collar size are presented as mean (SD). We finally performed a multiple linear regression analysis to identify correlations (r) between the independent predictors (age, gender, BMI, driving licence duration and collar size) with the dependent variable (ESS). A *p* value <0.05 was assumed to represent statistical significance.

## Results

### Demographics

One hundred thirty-eight patients were screened for the study, but 14 patients did not meet the eligibility criteria and 2 did not complete the assessment (Fig. 1). A total of 122 patients were included in the data analysis (72 males, age 59.4 years (15.2), BMI 32.1 kg/m^2^ (8.3), collar size 42.7 cm (5.0), smoking (*n* = 20): pack years 15 (10–33); ex-smoking (*n* = 49): pack years 20 (10–40); alcohol (n-62): 16 (4.75–40) units per week). Female participants were slightly younger, smaller and lighter, although their BMI was about the same as that of male participants; men smoked more and had a larger neck circumference (Table 1). Ninety-four patients held a driving licence for 25.2 years (20.6), and their age was 59.6 years (13.9). The longer patients held their driving licence, the older they were (r = 0.387, *p* < 0.001). There was a negative correlation between their age and the ESS (r = − 0.334, *p* < 0.001), as well as between the duration of holding a driving licence and the ESS (r = − 0.363, *p* < 0.001).

**Table 1:** Demographics of the studied cohort of patients

Parameter	Total (*n* = 122)	Female (*n* = 50)	Male (*n* = 72)
Age (years)	59.4 (15.2)	57.6 (17.6)	60.8 (13.9)
Height (m)	1.70 (0.12)	1.59 (0.09)	1.77 (0.08)
Weight (kg)	92.5 (28.8)	78.8 (23.8)	101.6 (28.3)
BMI (kg/m^2^)	32.1 (8.3)	31.0 (8.6)	32.6 (8.3)
Neck circumference (cm)	42.7 (5.0)	40.1 (5.0)	44.0 (4.5)
Smoking status (never, *n*/pack years)	*n* = 61/25.7 (25.7)	*n* = 34/19.3 (17.6)	*n* = 27/30.0 (29.0)

Sleep-related breathing disorders were present in 53.0% of the patients (patients were naïve to continuous positive airway pressure, CPAP, at the time of assessment), 28.0% of participants had neuromuscular disorders with associated chest wall disease (NMD/CWD), 17.0% had obstructive airway disorders, hypersomnias in 14.0%, sleep movement disorders were present in 1.0%, parasomnias in 1.0% and 18.0% of participants were classified as ‘other’ (this group consisted of patients with depression, anxiety, cardiac comorbidity, seizures, diabetes, hypothyroidism or unexplained syncopes). 65.6% of patients identified as White British, 4.9% Black British, 6.6% Black African, 6.6% Black Caribbean and 16.4% classified as other. Thirty-seven patients reported having an allergy but no patient reported any previous RTAs; three patients reported the use of legal highs/illegal substances.

### Sleepiness assessments

**Fig. 2:**
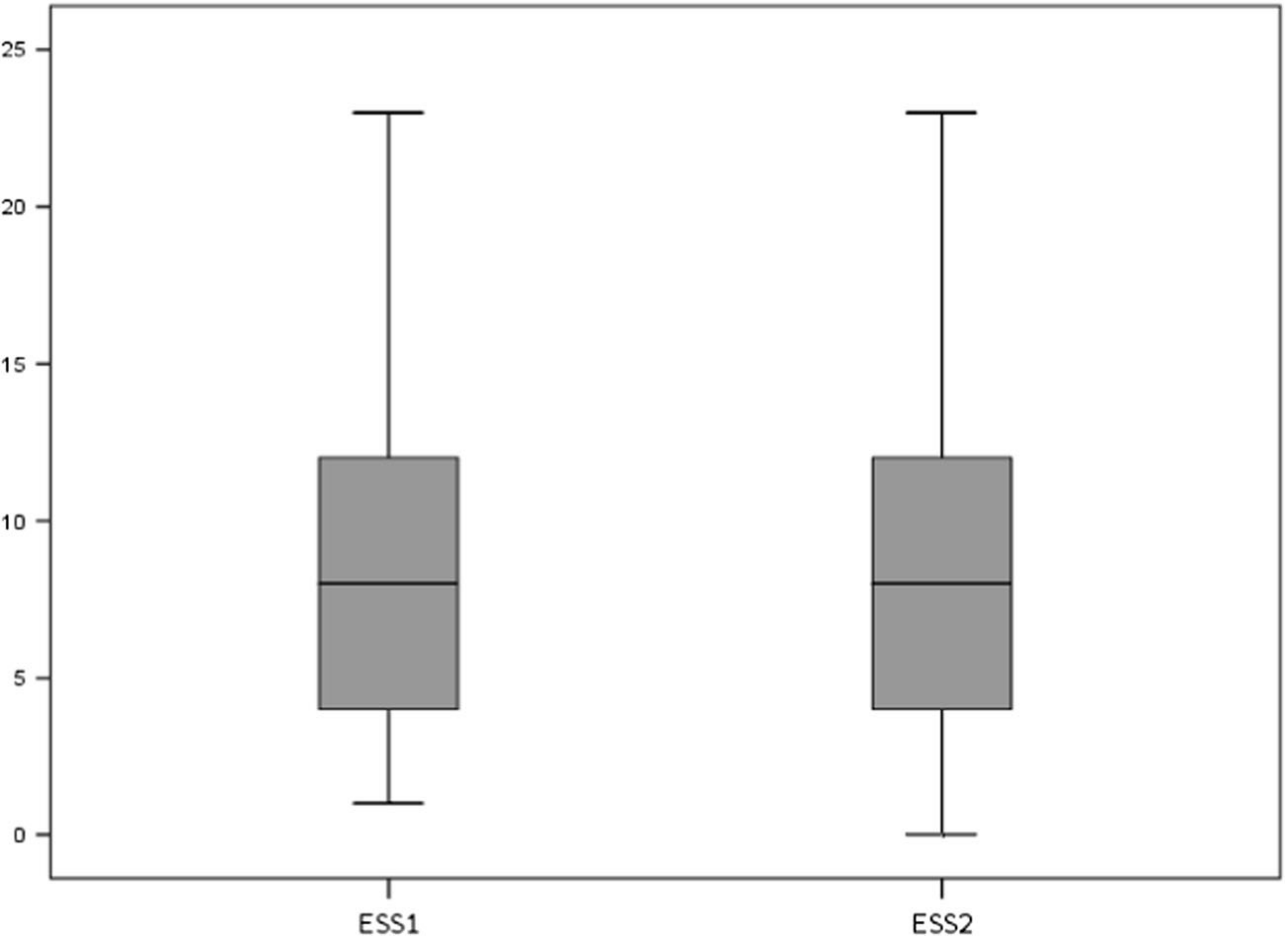
Box-Whisker plot for the Epworth Sleepiness Scale (ESS), first (ESS1) vs second score (ESS2), *p* = 0.289

**Fig. 3:**
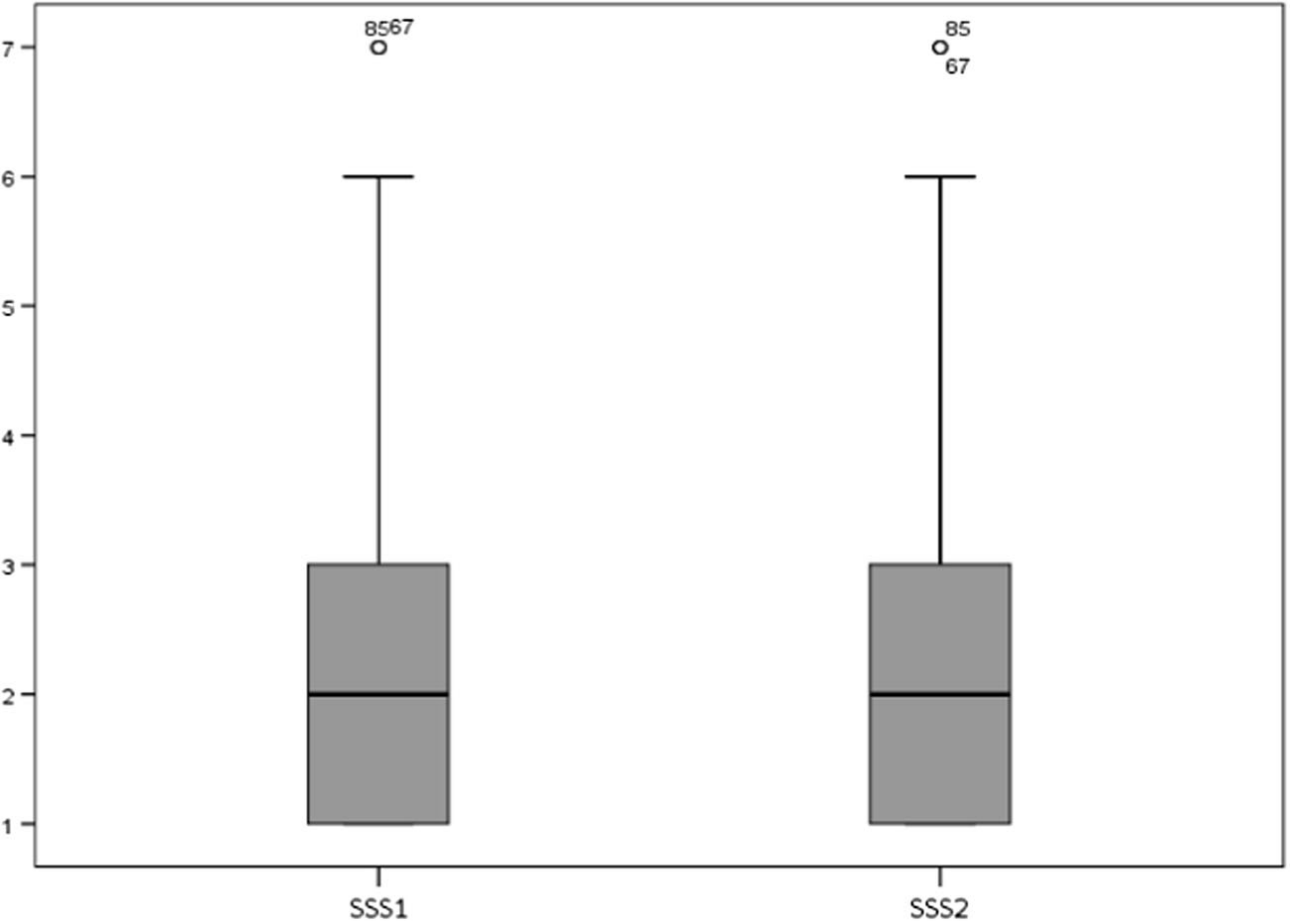
Box-Whisker plot for the Stanford Sleepiness Scale (SSS), first (SSS1) vs second (SSS2) scores, *p* = 0.320

There was no significant difference in the ESS [8 (8) vs 8 (8) points; *p* = 0.289] (Fig. 2) or the SSS [2 (2.25) vs 2 (2) points; *p* = 0.320] between first and second scores (Fig. 3). A total of 39 sleepy and 83 non-sleepy patients were identified, based on the ESS cutoff (> 10 points). There was no change between the scores in the ESS (*p* = 0.430) or the SSS (*p* = 0.830) based on subgroup analysis (sleepy/non-sleepy) either. A total of seven patients (5.7%) changed their scores from “sleepy” to “non-sleepy,” and four patients (3.3%) changed from non-sleepy to sleepy during their second assessments. A total of 35 patients reported significant changes in the ESS scores of more than 1 point (28.7%) and 20 patients by more than 2 points (16.4%). For the total cohort, the change in the ESS from baseline was − 0.11 (2.67) points, and for the SSS, the change was − 0.04 (0.42) points.

A stepwise multiple linear regression analysis to predict the average ESS scores included age, gender, BMI, driving licence duration, smoking pack years and collar size as independent variables (alpha = 0.05). Driving licence holder duration (*p* = 0.009) and age (*p* = 0.008) were identified as independent predictors of the ESS, while gender (*p* = 0.990), BMI (*p* = 0.697), smoking history (*p* = 0.95) and collar size (*p* = 0.812) were excluded from the model. About 17% of the variability of the ESS score were accounted for by this model (adjusted R^2^ = 0.171, F(6,112) = 5.043, *p* < 0.05).

## Discussion

Patients attending sleep clinics who are provided with information about sleepiness and fitness-to-drive provide largely reproducible information when reporting their symptoms using standard tools like the Epworth or the Stanford Sleepiness Scale. Despite high intra-individually variability, only 9.0% of the patients change the Epworth Sleepiness Scale score in a relevant way, while 5.7% of patients re-consider and change their score from sleepy to non-sleepy. How long someone held their driving licence and how old they are accounted for about 17% of the observed variability in the Epworth Sleepiness Scale scores. There was no significant difference in the results that was related to subscores or other factors like RTA or duration of driving licence.

### Clinical significance

Self-reported questionnaires for the assessment of sleepiness are commonly used in sleep laboratories, and previous studies have identified internal consistency, reliability and validity of the ESS [11]. Current DVLA guidelines advocate that those who suffer from conditions resulting in excessive daytime sleepiness need to inform the DVLA and information regarding a patient’s condition may then be requested from medical health professionals [7].

In this study, the Epworth Sleepiness Scale was chosen as it is a widely used tool within sleep centres and accessible to patients with sleep disorders. In the context of fitness-to-drive, the Epworth Sleepiness Scale is referred to when writing medical reports for the Drivers and Vehicles Licensing Agency (DVLA) in the UK. However, the ESS typically does not respond to changes in sleepiness quickly. The Stanford Sleepiness Scale is more suitable to pick up ad hoc changes in alertness, as it can be used to assess hourly daytime alertness.

The results from this study support the general validity when using results from the patients’ ESS, as the information provided is not significantly influenced by additional information about the DVLA requirements. This rejects the idea that patients display significant and unconscious bias when self-reporting sleepiness [12], an observation that proves valuable in terms of assessing patient conditions associated with sleepiness, and when assessing fitness-to-drive. Although about 1/6 patients scored higher than what would be the expected minimally clinical important difference in the ESS [13], this only changed the ESS score in a relevant way in about 5% of the cases (non-sleepy to sleepy), which is about the expected level for random findings and errors for confidence intervals.

### Limitations

While efforts were made to standardise the length of time between patients completing their first ESS and being interviewed for the second ESS, this was not always possible. Although all assessments finished within a 30–60 min period, as such, these differences may account for some variation in how patients reported their sleepiness. Prior patient knowledge regarding the ESS and DVLA criteria may have further influenced results, as some patients may have previously heard about the DVLA. This may have also been influenced by the variation in duration of diagnoses, patient knowledge and expertise and lack of inclusion of additional information, e.g. about the educational background, which may have confounded results. Furthermore, the study did not include objective measures of sleepiness and relied solely upon subjective and self-reported measurements that are the current standard for sleep clinics. It may therefore prove beneficial to include other objective measures to further evaluate the impact on patients’ sleepiness and compare objective and subjective measures of sleepiness assessment.

### Conclusion

Self-assessment tools for sleepiness provide more robust and reproducible information than expected. Only in a minority of patients, there is a relevant change in the scores following provision of information about fitness-to-drive and clinicians should be encouraged to inform patients about potential implications prior to filling in the Epworth Sleepiness Scale.

## References

[1] Pagel J (2009). Excessive daytime sleepiness. Am Fam Physician.

[2] Slater G, Pengo MF, Kosky C, Steier J (2013). Obesity as an independent predictor of subjective excessive daytime sleepiness. Respir Med.

[3] Roth T (2015). Effects of excessive daytime sleepiness and fatigue on overall health and cognitive function. J Clin Psychiatry.

[4] Nabi H, Guéguen A, Chiron M, Lafont S, Zins M, Lagarde E (2006). Awareness of driving while sleepy and road traffic accidents: prospective study in GAZEL cohort. BMJ.

[5] de Mello MT, Narciso FV, Tufik S, Paiva T, Spence DW, Bahammam AS, Verster JC, Pandi-Perumal SR (2013). Sleep disorders as a cause of motor vehicle collisions. Int J Prev Med.

[6] Assessing fitness to drive: a guide for medical professionals - GOV.UK. 04/08/2017]; Available from: http://www.gov.uk/dvla/fitnesstodrive

[7] Tiredness can kill: advice for drivers. 17/09/2017]; Available from: https://www.gov.uk/government/uploads/system/uploads/attachment_data/file/503534/INF159_150216.pdf

[8] Johns MW (1991). A new method for measuring daytime sleepiness: the Epworth sleepiness scale. Sleep.

[9] Hoddes E, Zarcone V, and Dement W (1972) Development and use of Stanford sleepiness scale (SSS). In Psychophysiology. Cambridge Univ Press, New York

[10] Herscovitch J, Broughton R (1981). Sensitivity of the Stanford sleepiness scale to the effects of cumulative partial sleep deprivation and recovery oversleeping. Sleep.

[11] Spira AP, Beaudreau SA, Stone KL, Kezirian EJ, Lui LY, Redline S, Ancoli-Israel S, Ensrud K, Stewart A, for the Osteoporotic Fractures in Men Study (2012). Reliability and validity of the Pittsburgh sleep quality index and the Epworth Sleepiness Scale in older men. J Gerontol A Biol Sci Med Sci.

[12] Nickerson RS (1998). Confirmation bias: a ubiquitous phenomenon in many guises. Rev Gen Psychol.

[13] Patel Suhani, Kon Samantha S. C., Nolan Claire M., Barker Ruth E., Simonds Anita K., Morrell Mary J., Man William D.-C. (2018). The Epworth Sleepiness Scale: Minimum Clinically Important Difference in Obstructive Sleep Apnea. American Journal of Respiratory and Critical Care Medicine.

